# Supplementary material for: Investigating the Electromechanical Sensitivity of Carbon-Nanotube-Coated Microfibers
Source: Sensors (Basel). 2023 May 30;23(11):5190. doi: 10.3390/s23115190 (PMC10255525; doi:10.3390/s23115190)
Supplement: Supplementary file 1 [file sensors-23-05190-s001.zip › sensors-2352775-supplementary.pdf]

sensors

MDPI

Supplementary Material

Investigating the Electromechanical Sensitivity of Carbon-Nanotube-Coated Microfibers

Elizabeth Bellott [1], Yushan Li [1], Connor Gunter [2], Scott Kovaleski [2] and Matthew R. Maschmann [1,3,*]

1 Department of Mechanical & Aerospace Engineering, University of Missouri, Columbia, MO 65211, USA
2 Department of Electrical Engineering & Computer Science, University of Missouri, Columbia, MO 65211, USA
3 MU Materials Science and Engineering Institute, University of Missouri, Columbia, MO 65211, USA
* Correspondence: maschmannm@missouri.edu

1. Additional CNT Forest-Coated Fiber Images

The acquisition of CNT outer diameters was accomplished using a ThermoFisher Pharos G2 field emission scanning electron microscopy (FE-SEM). The measurements were acquired using the TermoFisher ProSuite software package and compiled to make histograms. The images and measurements from which the histograms shown in Figure 3 of the manuscript are shown in Figure S1.

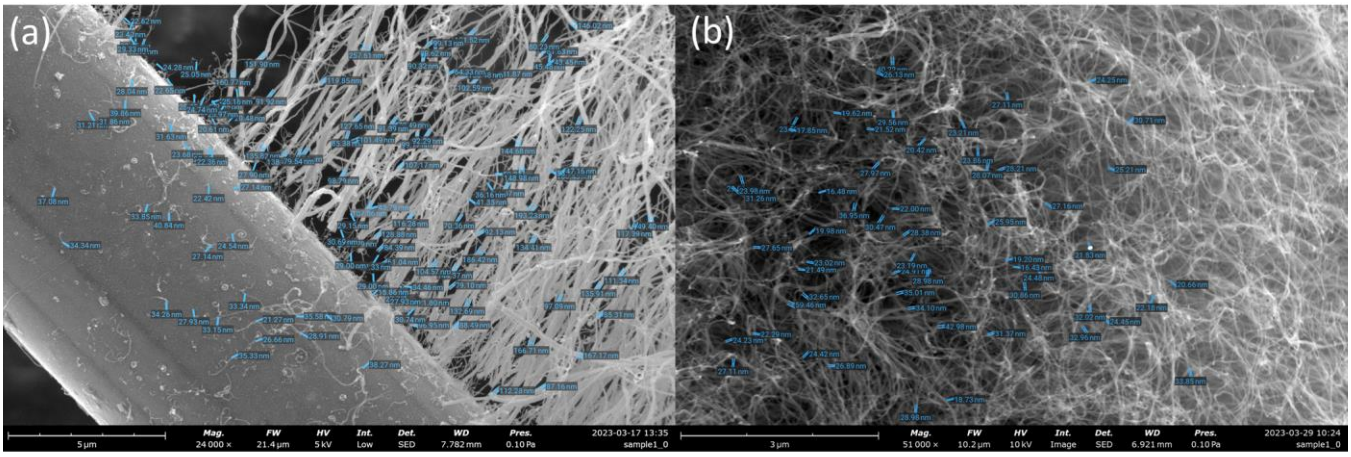

Figure S1. SEM measurements of CNT diameters from (a) as-received glass fibers and (b) alumina-coated glass fibers.

An additional SEM image showing the formation of two CNT forest bands originating radially from the fiber substrate is shown in Figure S2.

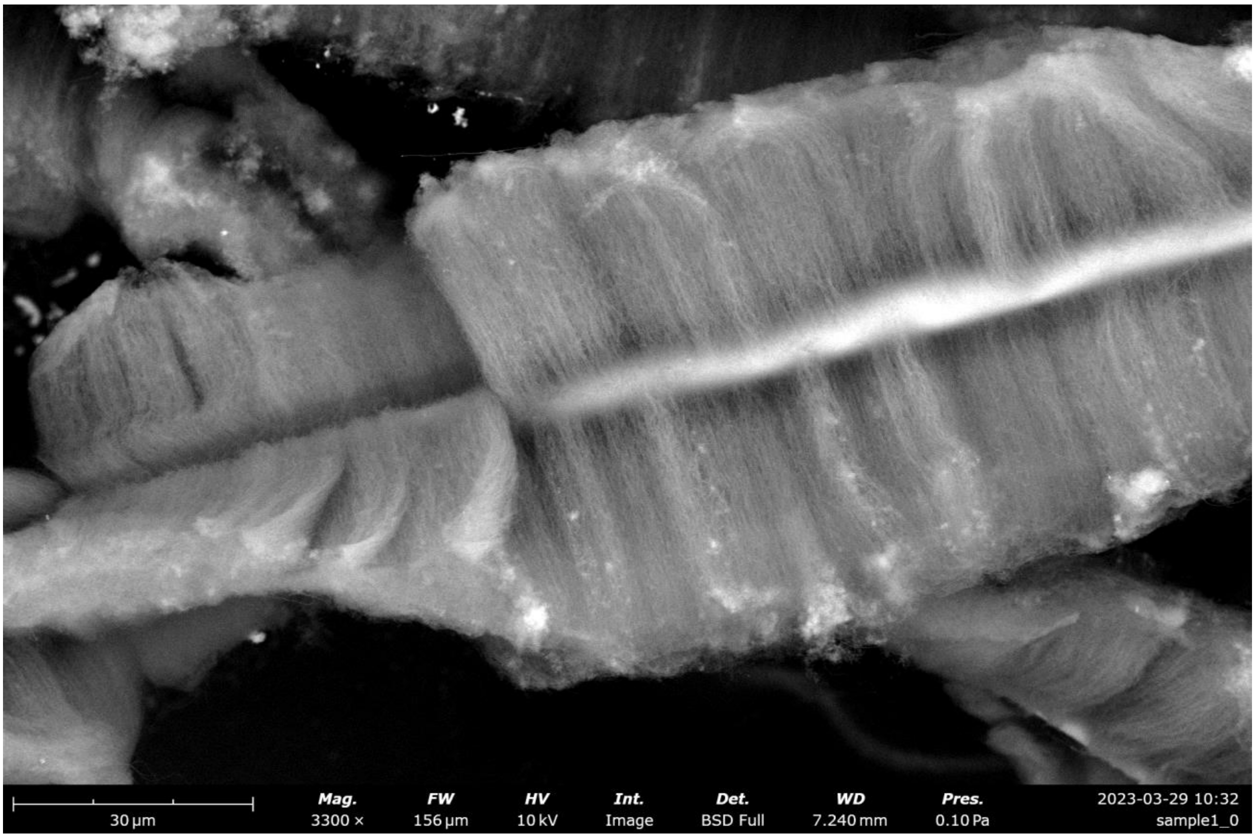

Figure S2. SEM image showing a CNT forest-coated glass fiber with two dense bands of CNTs.

2. CNT Simulation Output

The electrical resistance of CNT forests was simulated using a custom finite element code, as described in the manuscript. The total electrical resistance of each forests represents the sum of the electrical contact resistance between the CNTs and the electrodes and the intrinsic resistance of the CNT forests. The contact resistance is defined as the resistance between CNT nodes and the electrodes. The intrinsic resistance is defined as the resistance established within the CNT forest if the CNT nodes contacting an electrode surface were at the same potential as the electrodes themselves. In the simulation, each CNT-CNT contact resulted in a conduction pathway between CNTs with a resistance of 50 kΩ. Each node in contact with the measurement electrode established a conductive pathway with a resistance of 10 kΩ. The resistance of a given CNT is negligible compared to these values. The total contact resistance and the intrinsic internal CNT forest resistance as a function of CNT-electrode contact and CNT-CNT contacts is shown in Figure S3. Note that the intrinsic resistance is computed when the CNT nodes in contact with an electrode are assumed to be at the same potential as the electrodes themselves. A such, the total resistance is the sum of the contact resistance and the intrinsic resistance.

In the simulations, the maximum contact resistance allowed is 20 kΩ, representing one node in contact with the left electrode and one in contact with the right electrode. While the 10 nm O.D. CNT forests did not achieve a resistance of this magnitude, most of the 40 nm O.D. CNT forests (Figure S3c) simulated did obtain an initial value of 20 kΩ,. Because of the increased density associated with the 10 nm diameter CNTs, three or more electrical contacts were established at first contact, whereas the lower density associated with the 40 nm O.D. CNT forests facilitated just one contact point per electrode. In all cases, the electrical resistance was inversely related to the number of CNT-electrode or CNT-CNT contact points. A precipitous drop in the intrinsic resistance of all forests observed at the lowest number of CNT-CNT contacts is more associated with the establishment of new CNT-electrode contacts than with an internal increase in CNT-CNT contact

points. The intrinsic resistance for each CNT length then achieves a smoothly decreasing trend with increased CNT-CNT contacts, as the quantity of new CNT-CNT contacts greatly exceeds that of new CNT-electrode contacts. The drastic reduction in resistance caused by an early onset of new CNT-electrode contacts, stressing that the formation of CNT-electrode contacts is the primary mechanism governing the piezoresistive response of compressed CNT forests.

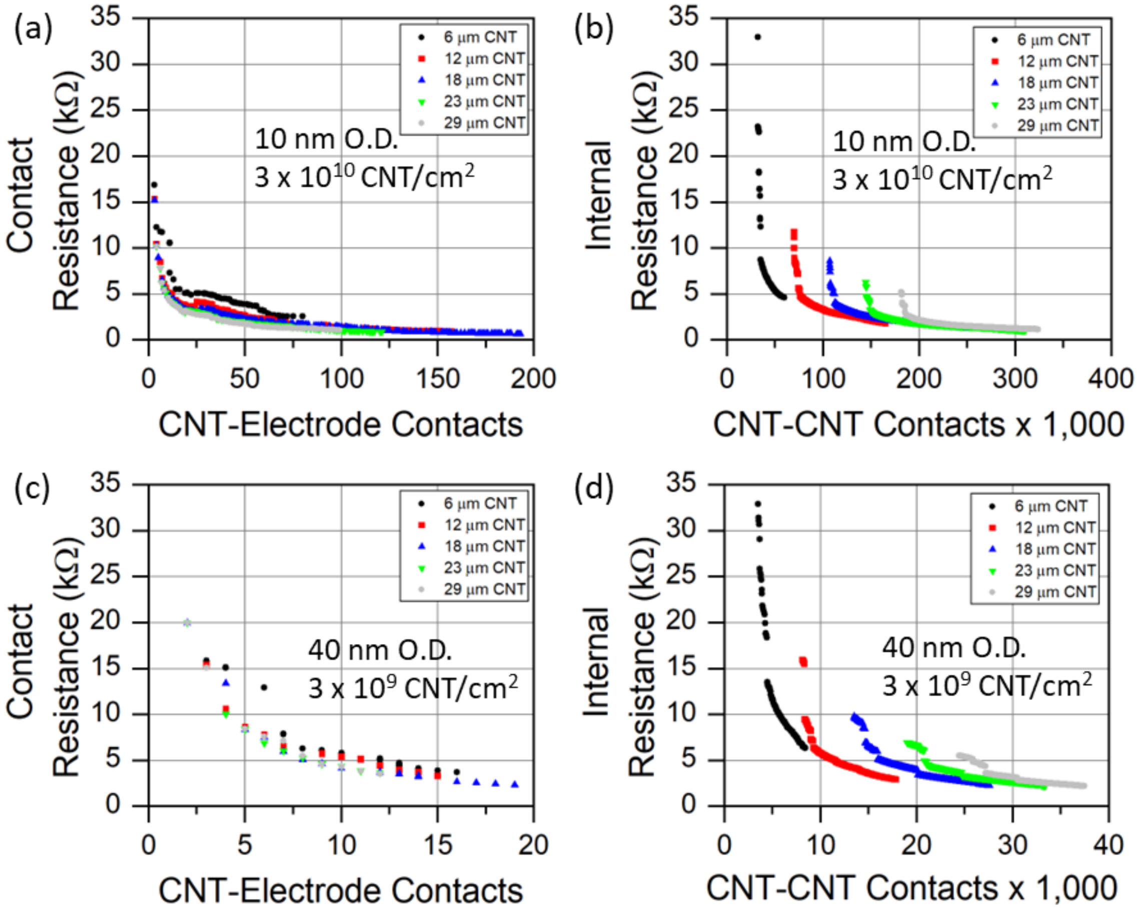

Figure S3. Simulated (a,c) contact resistance and (b,d) intrinsic internal resistance of compressed CNT forests. The top images (a-b) represent 10 nm outer diameter CNTs synthesized at a density of 3 x 10^{10} CNT/cm^2, while the bottom images (c-d) represent 40 nm outer diameter CNTs synthesized at a density of 3 x 10^9CNT/cm^2.
